# The Future of the Voluntary Hospitals

**Published:** 1922-04

**Authors:** 


### THE FUTURE OF THE VOLUNTARY HOSPITALS.

**By the Right Hon. the EARL OF ONSLOW, Chairman of the Voluntary Hospitals Commission.**

LAST year the Committee, under Lord Cave's chairmanship, appointed to consider the whole question of the voluntary hospitals, strongly recommended that everything should be done to save and maintain the voluntary system, and in this conclusion the Government were and are entirely and absolutely in accord. In all they have done in this matter the first principle in the mind of the Government has been that the voluntary system must be saved. But they felt that the voluntary system would soon cease to be a voluntary system if the hospitals were not asked to co-operate to raise new revenue required to meet necessary post-war expenditure. The voluntary system would be sorely damaged by the gift of unconditional deficiency grants from the Exchequer. Each year such grants were given would, with a certainty, cause voluntary effort to relax, subscriptions and money from sources other than the Treasury would fall off, the deficiency to be met by the State would grow; slowly, perhaps, at first, but then by leaps and bounds; and State or municipal control, the one thing which we wish to avoid, must inevitably follow. Therefore, when Lord Cave's Committee recommended that the whole deficit for 1921, estimated at about £1,000,000, should be made good from an Exchequer grant, the Government took the view that it was not fair to the heavily burdened taxpayer, nor was it in the interests of the hospitals themselves, to relieve them of all the burden of expanding their revenue. It was decided, therefore, that half a million pounds should be found from State funds, subject to the condition that the hospitals themselves, individually or collectively, should raise a corresponding amount.

A further recommendation was that a Voluntary Hospitals Commission should be set up and Local Hospital Committees established in the provinces. This Commission was appointed in July of last year.

When the Commission met they were faced with two immediate problems—the creation of these Local Committees, and the crisis which had arisen in connection with some of the London hospitals, who had closed beds or were in immediate danger of having to close them. Fortunately, there was machinery ready to hand for dealing with the latter emergency. King Edward's Hospital Fund for London undertook to act as the Local Voluntary Hospital Committee for the Metropolitan Area, and furnished the Commission with reports on the position in London. As a result, the Commission were able early in November to make emergency grants to certain of the London hospitals amounting to £77,900. In December a grant of £10,000 was made to the London Hospital to enable them to claim a gift of £10,000 made by an American on condition that a similar sum was subscribed in England before December 31 last.

To return to the establishment of the Local Voluntary Hospital Committees. As a general rule the County with the County Boroughs in it has been taken as the unit on which a Voluntary Hospital Committee was based; but some of the largest County Boroughs have been constituted units by themselves. The constitution recommended for a Local Committee is as follows: Two representatives of the County Council, one representative of each County Borough, two medical practitioners nominated by the Medical Committee in the area, one being a general practitioner and the other on the staff of a voluntary hospital; two hospital representatives, one representing the larger and one the smaller hospitals in the area. The Commission reserved the right to nominate not more than five additional members from among those resident in the area, of whom at least one would be a woman. That is the normal basis on which the Committees have been constituted. But there have been many variations in details. There have been various groupings of areas, and modifications of the personnel of the Committees to suit local conditions and local preferences.

In some quarters the Committees have been criticised as including those who had not previously been associated with hospital work. I may say that this has been done deliberately. We want to widen the area of persons interested in hospitals and interested in the maintenance of the voluntary system, and to increase the support accorded to interest in and knowledge of the voluntary system.

In England Committees have now been practically established in 37 out of 46 areas; in Wales, in three out of four; and in Scotland, four out of 12.

Now, these Committees are not expected to control the hospitals in any way. It would be impossible to suggest that they should have any powers of compulsion. What we wish to do is to maintain the voluntary system, and to do that we must maintain the voluntary principle, and to introduce any element of compulsion or any direct authority over the hospitals would be to destroy the voluntary principle.

I am sometimes asked, "Will the Local Committees go on after the grant has been disposed of?" The answer seems to me to depend on the conditions prevailing when the grant has been distributed. But I venture to think that where the Committees have proved helpful and useful, and have won the confidence of the hospitals, the hospitals themselves will wish the Committees to continue.

To deal now with the functions which it has been suggested that the Voluntary Hospital Committees should perform. They have been described as follows in the Circular sent by the Commission to the Committees:—

(*a*) To act as local advisers to the Commission.
(*b*) To collect information as to the needs of their area.
(*c*) To further co-operation between hospitals.
(*d*) To co-ordinate appeals.
(*e*) To consider the possibility of arranging the transfer of patients where this can advantageously be done.

(*f*) To prepare, where practicable, schemes of co-operative purchase.
(*g*) To advise as to the adoption of a uniform system of hospital accounts throughout the area.
(*h*) To organise systematic contributions both from employers and employees in areas where no such systems at present exist.
(*i*) To undertake the distribution of any contributions made by an Approved Society in cases where the society is purely local.

In assisting in co-ordinating appeals it is hoped the Committee will recognise the great possibilities of the small regular contributions. Hitherto the main reliance has been placed on big cheques and big legacies, but the small weekly contribution has hitherto not been developed to the extent to which it is believed it may in the future attain. An instance of what is being done in this direction may be cited in the recent decision of King Edward's Fund and also in Sheffield.

Another source of revenue is from contributions of the Approved Societies, and I am glad to say that the National Deposit, Prudential and the National Amalgamated Approved Societies and others have agreed to a scheme whereby their members coming into hospital will be relieved of the necessity of payment, or of contributions asked for by the almoner, up to the amount of the societies' contribution. It is expected that this will bring in a sum of £100,000 a year to the hospitals. Of course, all of it will not be new money, as part of it will go in reductions of payment which would otherwise have been met by the patients themselves. This generous and far-sighted scheme, however, which is fully approved of by the Ministry of Health, does represent a large amount of new money.

Then the question of the provision of hospital accommodation requires careful examination. There are a number of beds in Poor Law infirmaries constantly vacant, and it is possible that some scheme might be evolved whereby these beds might be made available for the accommodation of patients.

The Poor Law infirmaries and the beds therein are the property of the Guardians and of the ratepayers. Without their wish and consent nothing can be done, and with them the decision lies. But, in the first instance, the Voluntary Committees can examine into the hospital situation in their areas, and through their medium perhaps schemes may be devised for co-operation with the Guardians.

I now come to the methods by which the Committees may serve in co-ordinating the activities of the hospitals in saving money. It is clear from the experience already gained in London, that great advantage results from a uniform system of accounting. Therefore, we have appointed a Committee to advise as to the adoption of such a system. This Committee is in close contact with a similar Committee appointed by King Edward's Fund.

The other point on which I wish to touch as regards the assistance which the Committees may perhaps afford to hospitals is that of arranging systems of transfer from one hospital to another. There are many hospitals which have long waiting lists for acute cases, and it is found that the beds are blocked by chronic cases which could be equally well treated, if not better treated, in some smaller institution adapted more for after-care than for treatment, and such hospitals may have beds unoccupied and no waiting list. The Committees may, I think, organise schemes for the transfer of patients from one hospital to another within their area. In many counties, owing to the efforts of the Order of St. John of Jerusalem and the British Red Cross Society, there exist facilities for ambulance transport, and a general linking-up of all the services between hospitals and ambulances within the area should be greatly to the advantage of all concerned.

I have already mentioned the amount of money which has been granted to the London hospitals, and perhaps it may be asked in what way assistance is being given to provincial hospitals. The deficit of London is estimated by King Edward's Fund for last year to be £360,000. The moiety of that is £180,000, which would be due from the Government grant; and of that, about £90,000 has already been expended, so there remains a sum of £90,000 on our present calculation, which may be required for London. This leaves a sum of £320,000 for the rest of the work of the Commission. I may say that the £360,000 estimated for the London deficit is less than was budgeted for by Lord Cave, and so possibly more money may be available for the share of London, but I cannot say anything more about that until the accounts of all hospitals are complete.

As regards the provinces, very few applications have yet been received. I think that the reason for this is that the hospitals, before sending in their demands, are waiting to complete their annual accounts, and we expect, therefore, to have a large number of demands at the beginning of the next financial year, but we do not anticipate spending more than £120,000 before the end of this financial year.

I cannot help saying, before I conclude, that the outlook for the future of the voluntary system is increasingly encouraging. Although at present the difference between income and expenditure is wide, the gulf is not so wide as it has been. Falling prices are mainly responsible for this, but it is also to be noted that in spite of unemployment the income of the hospitals is on the whole maintained, and in many cases it is even increased.

If the supporters of the voluntary system themselves help in the full spirit of their splendid tradition, and if the community contribute their full share, I feel that what has been done for the hospitals should be adequate to save the voluntary system, especially if the fall in the cost of living continues. I am confident that if the public and the hospitals will co-operate in the future as they have done in the past, the system will withstand the present strain, and being established on a stronger foundation, will continue to keep the hospitals of this country in the forefront of progress. In this great task all the sympathy and all the help which is within the power of the Ministry of Health will be freely at their disposal as it has been throughout the crisis.

